# Supplementary material for: Amelioration of Brain Damage after Treatment with the Methanolic Extract of Glycyrrhizae Radix et Rhizoma in Mice
Source: Pharmaceutics. 2022 Dec 12;14(12):2776. doi: 10.3390/pharmaceutics14122776 (PMC9781260; doi:10.3390/pharmaceutics14122776)
Supplement: Supplementary file 1 [file pharmaceutics-14-02776-s001.zip › Figures S1-S3.pdf]

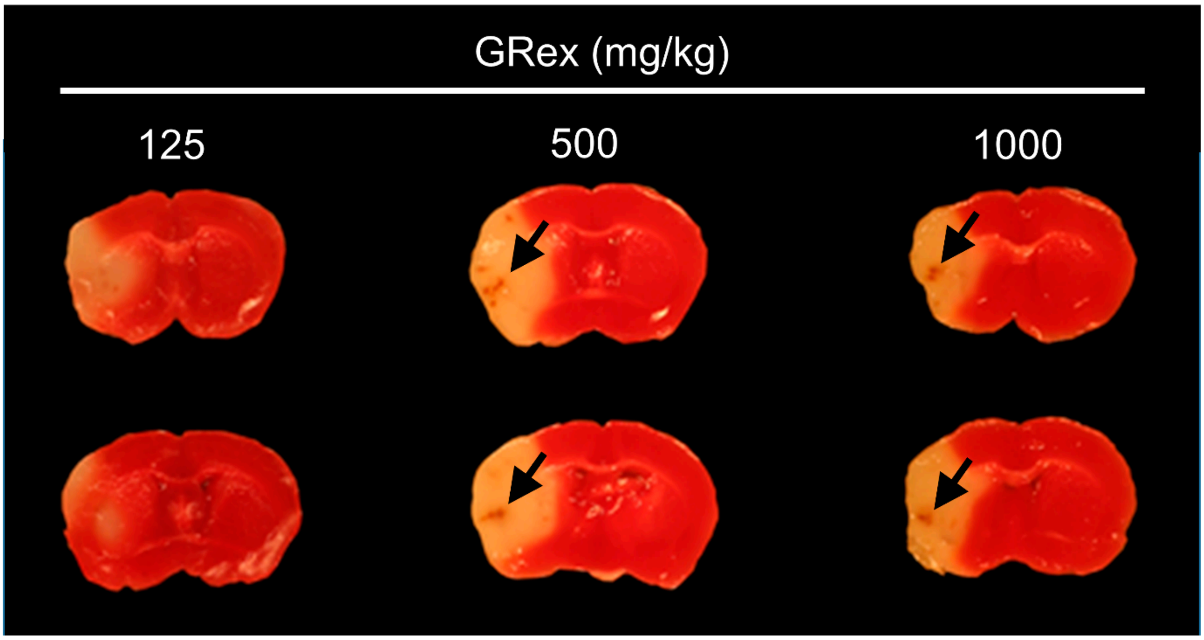

Figure S1. TTC-stained brain slice showing hemorrhagic transformation by GRex administration in MCAO mice model. Black arrows indicate dot-like microbleedings in 500 mg/kg or 1000 mg/kg GRex administered mice.

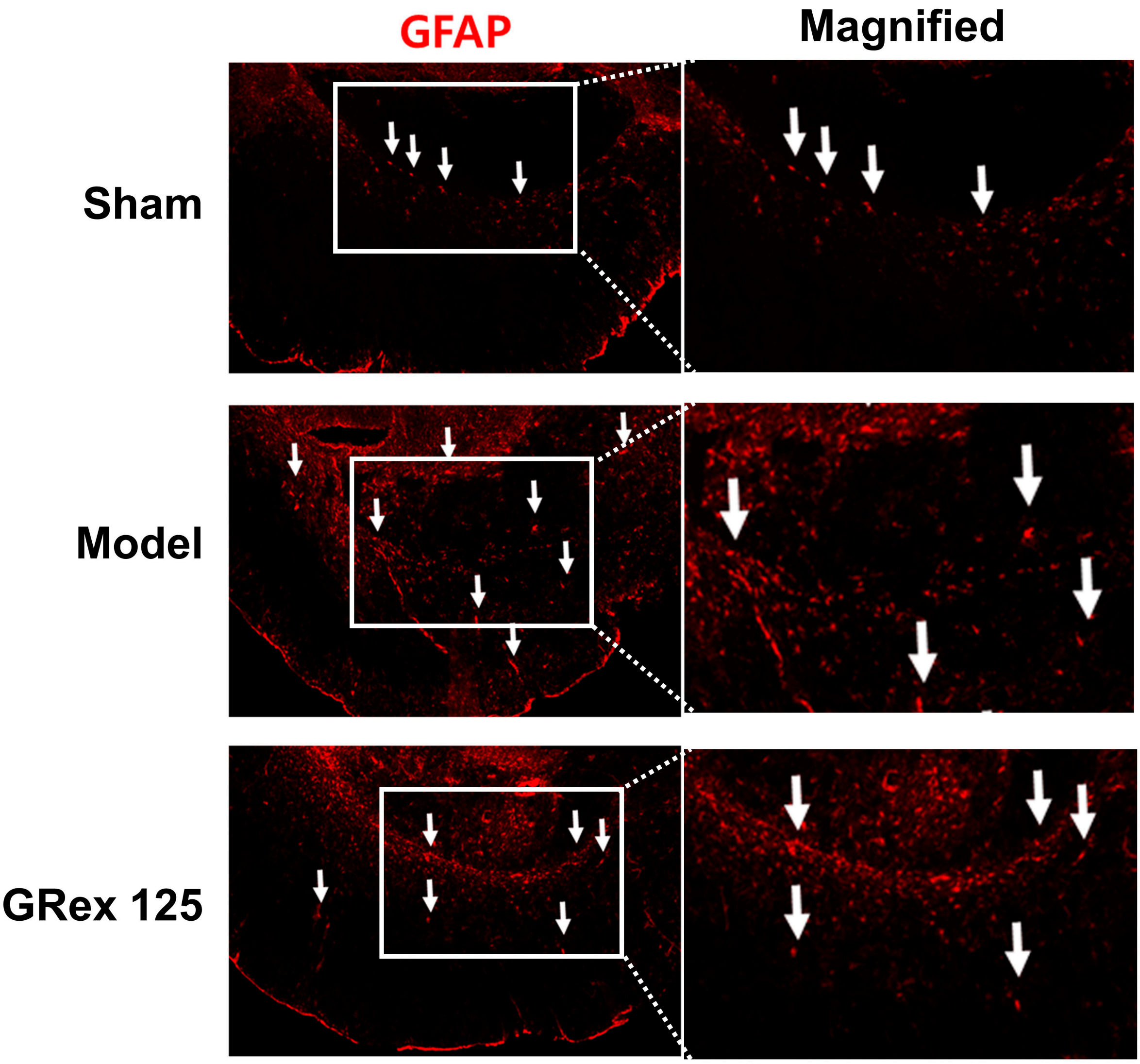

Figure S2. Inhibitory effect of GRex on GFAP expression in the cortical area of ischemic ipsilateral hemispheres. Each photomicrograph represents IF-stained cortical regions. White arrows indicate GFAP positive activated astrocytes.

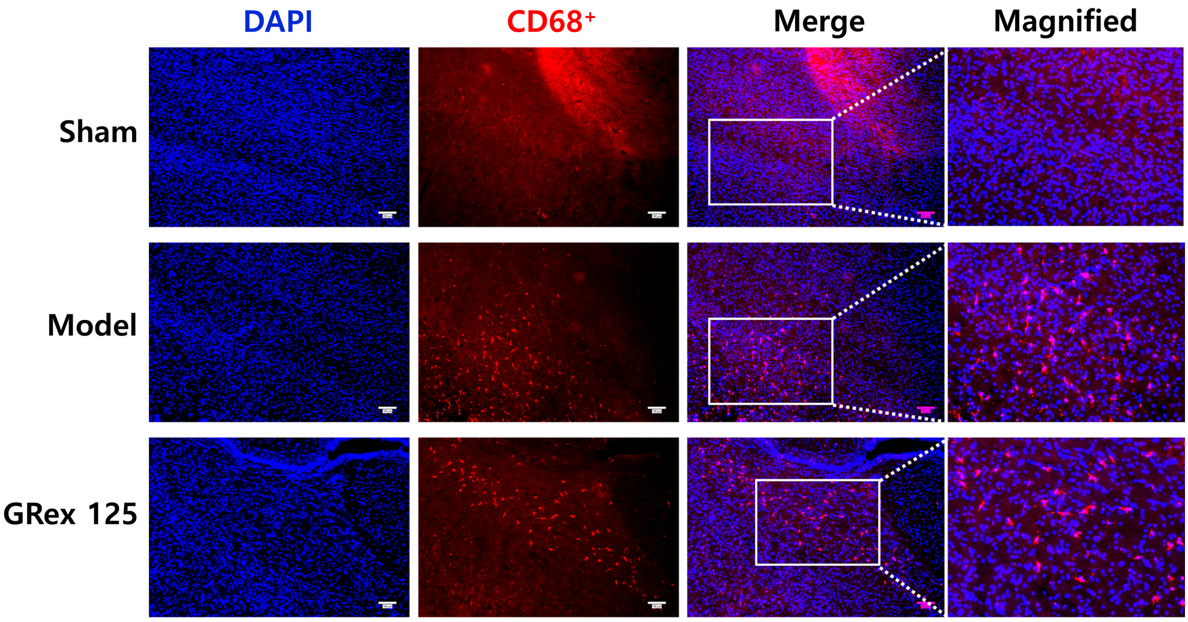

Figure S3. Inhibitory effect of GRex on CD68 expression in the cortical area of ischemic ipsilateral hemispheres. Each photomicrograph represents IF-stained cortical regions. White rectangle area indicate CD68 positive activated microglia.
