# Supplementary material for: Amelioration of Brain Damage after Treatment with the Methanolic Extract of Glycyrrhizae Radix et Rhizoma in Mice
Source: Pharmaceutics. 2022 Dec 12;14(12):2776. doi: 10.3390/pharmaceutics14122776 (PMC9781260; doi:10.3390/pharmaceutics14122776)
Supplement: Supplementary file 1 [file pharmaceutics-14-02776-s001.zip › Table S1.pdf]

Table S1. Mol ID, chemical name, and molecular weight of 69 active compounds from Glycyrrhizae Radix et Rhizoma (GR) and their corresponding predicted OB, Caco-2, BBB, and DL scores.

Mol ID	Chemical name	MW	OB (%)	Caco-2	BBB	DL
MOL000211	Mairin	456.78	55.38	0.73	0.22	0.78
MOL000239	Jaranol	314.31	50.83	0.61	−0.22	0.29
MOL000263	Oleanolic acid	456.78	29.02	0.59	0.07	0.76
MOL000359	Sitosterol	414.79	36.91	1.32	0.87	0.75
MOL000392	Formononetin	268.28	69.67	0.78	0.02	0.21
MOL000467	Castanin	298.31	23.54	0.77	−0.1	0.27
MOL000497	Licochalcone a	338.43	40.79	0.82	−0.21	0.29
MOL000500	Vestitol	272.32	74.66	0.86	0.3	0.21
MOL001484	Inermine	284.28	75.18	0.89	0.4	0.54
MOL001792	DFV	256.27	32.76	0.51	−0.29	0.18
MOL002311	Glycyrol	366.39	90.78	0.71	−0.2	0.67
MOL002565	Medicarpin	270.3	49.22	1	0.53	0.34
MOL003656	Lupiwighteone	338.38	51.64	0.68	−0.23	0.37
MOL003896	7-Methoxy-2-methyl isoflavone	266.31	42.56	1.16	0.56	0.2
MOL004805	(2S)-2-[4-hydroxy-3-(3-methylbut-2-enyl)phenyl]-8,8-dimethyl-2,3-dihydropyrano[2,3-f]chromen-4-one	390.51	31.79	1	0.25	0.72
MOL004806	Euchrenone	406.56	30.29	1.09	0.39	0.57
MOL004808	Glyasperin B	370.43	65.22	0.47	−0.09	0.44
MOL004810	Glyasperin F	354.38	75.84	0.43	−0.15	0.54
MOL004811	Glyasperin C	356.45	45.56	0.71	0.07	0.4
MOL004814	Isotrifoliol	298.26	31.94	0.53	−0.25	0.42
MOL004815	(E)-1-(2,4-dihydroxyphenyl)-3-(2,2-dimethylchromen-6-yl)prop-2-en-1-one	322.38	39.62	0.66	−0.12	0.35
MOL004820	Kanzonols W	336.36	50.48	0.63	0.04	0.52
MOL004828	Glepidotin A	338.38	44.72	0.79	0.06	0.35
MOL004829	Glepidotin B	340.4	64.46	0.46	−0.09	0.34
MOL004833	Phaseolinisoflavan	324.4	32.01	1.01	0.46	0.45
MOL004835	Glypallichalcone	284.33	61.6	0.76	0.23	0.19
MOL004838	8-(6-hydroxy-2-benzofuranyl)-2,2-dimethyl-5-chromenol	308.35	58.44	1	0.34	0.38
MOL004848	Licochalcone G	354.43	49.25	0.64	−0.04	0.32
MOL004849	3-(2,4-dihydroxyphenyl)-8-(1,1-dimethylprop-2-enyl)-7-hydroxy-5-methoxy-coumarin	368.41	59.62	0.4	−0.23	0.43
MOL004855	Licoricone	382.44	63.58	0.53	−0.14	0.47
MOL004856	Gancaonin A	352.41	51.08	0.8	0.13	0.4
MOL004857	Gancaonin B	368.41	48.79	0.58	−0.1	0.45
MOL004863	3-(3,4-dihydroxyphenyl)-5,7-dihydroxy-8-(3-methylbut-2-enyl)chromone	354.38	66.37	0.52	−0.13	0.41
MOL004864	5,7-dihydroxy-3-(4-methoxyphenyl)-8-(3-	352.41	30.49	0.9	0.21	0.41

	methylbut-2-enyl)chromone					
MOL004866	2-(3,4-dihydroxyphenyl)-5,7-dihydroxy-6-(3-methylbut-2-enyl)chromone	354.38	44.15	0.48	−0.28	0.41
MOL004879	Glycyrin	382.44	52.61	0.59	−0.13	0.47
MOL004882	Licocoumarone	340.4	33.21	0.84	0.06	0.36
MOL004883	Licoisoflavone	354.38	41.61	0.37	−0.27	0.42
MOL004884	Licoisoflavone B	352.36	38.93	0.46	−0.18	0.55
MOL004885	Licoisoflavanone	354.38	52.47	0.39	−0.22	0.54
MOL004891	Shinpterocarpin	322.38	80.3	1.1	0.68	0.73
MOL004907	Glyzaglabrin	298.26	61.07	0.34	−0.2	0.35
MOL004908	Glabridin	324.4	53.25	0.97	0.36	0.47
MOL004910	Glabranin	324.4	52.9	0.97	0.31	0.31
MOL004911	Glabrene	322.38	46.27	0.99	0.04	0.44
MOL004912	Glabrone	336.36	52.51	0.59	−0.11	0.5
MOL004913	1,3-dihydroxy-9-methoxy-6-benzofurano[3,2-c]chromenone	298.26	48.14	0.48	−0.19	0.43
MOL004915	Eurycarpin A	338.38	43.28	0.43	−0.06	0.37
MOL004941	(2R)-7-hydroxy-2-(4-hydroxyphenyl)chroman-4-one	256.27	71.12	0.41	−0.25	0.18
MOL004945	(2S)-7-hydroxy-2-(4-hydroxyphenyl)-8-(3-methylbut-2-enyl)chroman-4-one	324.4	36.57	0.72	−0.04	0.32
MOL004948	Isoglycyrol	366.39	44.7	0.91	0.05	0.84
MOL004957	HMO	268.28	38.37	0.79	0.25	0.21
MOL004959	1-Methoxyphaseollidin	354.43	69.98	1.01	0.48	0.64
MOL004966	3'-Hydroxy-4'-O-Methylglabridin	354.43	43.71	1	0.73	0.57
MOL004974	3'-Methoxyglabridin	354.43	46.16	0.94	0.47	0.57
MOL004978	2-[(3R)-8,8-dimethyl-3,4-dihydro-2H-pyrano[6,5-f]chromen-3-yl]-5-methoxyphenol	338.43	36.21	1.12	0.61	0.52
MOL004980	Inflacoumarin A	322.38	39.71	0.73	−0.24	0.33
MOL004988	Kanzonol F	420.54	32.47	1.18	0.56	0.89
MOL004989	6-prenylated eriodictyol	356.4	39.22	0.4	−0.29	0.41
MOL004991	7-Acetoxy-2-methylisoflavone	294.32	38.92	0.74	0.16	0.26
MOL005000	Gancaonin G	352.41	60.44	0.78	0.23	0.39
MOL005001	Gancaonin H	420.49	50.1	0.6	−0.14	0.78
MOL005003	Licoagrocarpin	338.43	58.81	1.23	0.61	0.58
MOL005007	Glyasperins M	368.41	72.67	0.49	−0.04	0.59
MOL005012	Licoagroisoflavone	336.36	57.28	0.71	0.09	0.49
MOL005016	Odoratin	314.31	49.95	0.42	−0.24	0.3
MOL005017	Phaseol	336.36	78.77	0.76	−0.06	0.58
MOL005018	Xambioona	388.49	54.85	1.09	0.52	0.87
MOL005020	Dehydroglyasperins C	340.4	53.82	0.68	−0.12	0.37
