# Supplementary material for: Amelioration of Brain Damage after Treatment with the Methanolic Extract of Glycyrrhizae Radix et Rhizoma in Mice
Source: Pharmaceutics. 2022 Dec 12;14(12):2776. doi: 10.3390/pharmaceutics14122776 (PMC9781260; doi:10.3390/pharmaceutics14122776)
Supplement: Supplementary file 1 [file pharmaceutics-14-02776-s001.zip › Table S2.pdf]

Table S2. The frequency degree with which compound is connected to diseases in target-disease network.

Compound ID	Degree	Compound ID	Degree	Compound ID	Degree
MOL003896	156	MOL004908	116	MOL004805	86
MOL000392	141	MOL004957	116	MOL004855	86
MOL005003	140	MOL004810	115	MOL004814	85
MOL004959	139	MOL005020	114	MOL004945	83
MOL004991	137	MOL004857	112	MOL004829	82
MOL000500	133	MOL004864	112	MOL002311	80
MOL004974	132	MOL004915	112	MOL004941	78
MOL000467	131	MOL005000	112	MOL001484	77
MOL000497	129	MOL004856	111	MOL004910	75
MOL004978	129	MOL004912	111	MOL000239	73
MOL004828	128	MOL004883	110	MOL001792	73
MOL004966	127	MOL004820	108	MOL005001	72
MOL002565	126	MOL004884	106	MOL004913	68
MOL004811	126	MOL005012	105	MOL004989	64
MOL004835	126	MOL004815	104	MOL004806	57
MOL004891	125	MOL004848	104	MOL004948	57
MOL005007	122	MOL004863	104	MOL004988	56
MOL004833	120	MOL005016	104	MOL004838	55
MOL003656	119	MOL004866	103	MOL005018	51
MOL004911	118	MOL004980	102	MOL004882	46
MOL004808	116	MOL005017	102	MOL000359	6
MOL004849	116	MOL004907	101	MOL000263	4
MOL004885	116	MOL004879	90	MOL000211	1
