# Supplementary material for: Amelioration of Brain Damage after Treatment with the Methanolic Extract of Glycyrrhizae Radix et Rhizoma in Mice
Source: Pharmaceutics. 2022 Dec 12;14(12):2776. doi: 10.3390/pharmaceutics14122776 (PMC9781260; doi:10.3390/pharmaceutics14122776)
Supplement: Supplementary file 1 [file pharmaceutics-14-02776-s001.zip › Table S3.pdf]

Table S3. The frequency degree with which disease is connected to compounds in target-disease network.

Disease	Degree	Disease	Degree
Breast cancer	68	Pain, unspecified	64
Cancer, unspecific	67	Pathological angiogenesis	64
Alzheimer's Disease	66	Peutz-Jeghers syndrome	64
Cardiovascular disease, unspecified	66	Pyresis	64
Myocardial Infarction	66	Stroke	64
Prostate cancer	66	Vascular lesion regression	64
Bladder cancer	65	Cardiovascular disease	63
Inflammation	65	Endocrine independent cancer	63
Renal Cell Carcinoma	65	Migraine	63
Rheumatoid arthritis, unspecified	65	Osteoporosis	63
Abdominal aortic aneurysm	64	Osteoporosis, unspecified	63
Adenomatous polyposis	64	Postmenopausal symptoms	63
Analgesics	64	Solid tumors	61
Arthritis	64	Multiple Myeloma	59
Brain injury	64	Non-small Cell Lung Cancer	59
Carcinoma in situ, unspecified	64	Thrombosis	58
Carpal tunnel syndrome	64	Chronic inflammatory diseases	57
Colorectal cancer	64	Ischemia reperfusion injuries	55
Coronary atherosclerosis	64	Asthma	54
Dysmenorrhea, unspecified	64	Multiple Sclerosis	54
Endometriosis	64	Androgen insensitivity	53
Genitourinary tumors	64	Hypospadias 1, X-linked	53
Gestational hypertension	64	Spinal and bulbar muscular atrophy	53
Inflammatory diseases	64	Spinal and bulbar muscular atrophy of Kennedy	53
Lung Cancer	64	XY disorders of sex development (Disorders in androgen synthesis or action)	53
Malignant mesothelioma	64	XY disorders of sex development (Other)	53
Meningioma	64	Autoimmune Diseases	52
Neurodegenerative diseases	64	Diabetes mellitus	52
Oropharyngeal squamous cell carcinoma	64	Noninsulin-dependent diabetes mellitus	52
Osteoarthritis	64	Obesity	52

Disease	Degree	Disease	Degree
Crohns's Disease, unspecified	50	B-cell malignancies	45
Insulin resistance	50	Chronic lymphocytic leukemia (CLL)	45
Psoriasis	50	Hepatocellular Carcinoma (HCC)	45
Adrenocorticotrophic hormone-secreting pituitary tumors	49	Nasopharyngeal Cancer (NPC)	45
Atherosclerosis	49	Non-Hodgkin's Lymphoma	45
Atopic Dermatitis	49	Viral infection, unspecified	45
Coagulative disorders	49	Atrial fibrillation and flutter	43
Inflammatory Bowel Disease	49	Thromboembolism	43
Ischemic heart disease	49	Skin diseases	41
Pancreatic Cancer	49	Adult respiratory distress syndrome	40
Testicular cancer	49	Cardiac dysrhythmias	40
Thromboembolic disorders	49	Crescentic glomerulonephritis	40
Thrombotic disease	49	Cytokine-mediated diseases	40
Thyroid follicular carcinoma	49	Endotoxemia	40
Ulcerative colitis	49	Epileptic seizures	40
Chronic Myelogenous Leukemia (CML)	48	Pain	40
ER beta-positive prostate tumors	48	Refractory partial epilepsy	40
Gastrointestinal Stromal Tumors (GIST)	48	Sustained ventricular tachycardia	40
Hematological Malignancies	48	Cardiac arrhythmias	34
HER2-positive Metastatic Breast Cancer	48	Glaucoma	27
Melanoma	48	Malignancies	27
Ovarian cancer	48	Anxiety disorder, unspecified	26
Refractory Hematological Malignancies	48	Chronic obstructive pulmonary disease, unspecified	26
Vascular injury response	48	Depression	26
Ischemia	47	Gliomas	26
Bipolar Affective Disorder	46	Heparin-induced thrombocytopenia type II	26
Immunodeficiency	46	Multiple organ failure	26
Acute lymphoblastic leukemia (ALL)	45	Obstructive airway disease	26
Acute myeloid leukemia (AML)	45	Respiratory distress syndrome	26
Advanced solid tumors	45	Skeletal muscle wasting	26

Disease	Degree	Disease	Degree
Skeletal muscle weakness	26	Airway hyperreactivity	7
Bacterial Infections	25	Neurological diseases	7
Hypertension	25	Urge incontinence	7
Parkinson's disease	24	Major Depressive Disorder	6
Cognitive deficits	23	Cancers	4
Acute promyelocytic leukemia	22	Cough	4
Fungal diseases	22	Diarrhea	4
Herpes virus infection	22	Dyspnea	4
Leishmania Infections	22	Opioid-induced bowel dysfunction	4
Malaria	22	Anxiety Disorders	3
Trichomoniasis	22	Benign prostate hyperplasia	3
Schizophrenia	21	Drug dependence	3
Shy-Drager syndrome	20	Hypertrophic vascular disease	3
Blood group, Yt system	16	Insomnia	3
Hypoxic-ischemic encephalopathy	16	Neuropsychiatric disorders	3
Motor neurone disease	16	Opioid dependence	3
Parkinson Disease	15	Renal failure	3
Angiogenesis	14	Acute ureteric colic	2
Bronchial asthma	13	Arterial embolism and thrombosis	2
Bronchospasm (histamine induced)	13	Chemotherapy-induced nausea and vomiting	2
Chronic myeloid leukemia	13	Cocaine dependence	2
Vascular disease	13	Diabetic nephropathy	2
Angina	10	Diabetic neuropathy	2
Colon cancer	10	Essential (primary) hypertension	2
Coronary Artery Disease	10	Irritable bowel syndrome	2
Helminth infection	10	Leukemia, Myeloid	2
Hypertension, Angina	10	Manic disorder	2
Sepsis	10	Neurologic and psychiatric diseases	2
Angioedema	9	Oesophageal cancer	2
Heart Failure	9	Parkinsonian symptoms	2

Disease	Degree	Disease	Degree
Postoperative nausea and vomiting	2	Insulin-dependent diabetes mellitus	1
Allergic airway inflammation	1	Ischemic Stroke	1
Autoimmune and sudden sensorineural hearing loss	1	Metabolic Disease	1
Autoimmune cardiomyopathy	1	Metabolic syndrome X	1
Bronchoconstriction (cold air-induced)	1	Neurogenic bladder	1
Cancer (multidrug resistant)	1	Pancreatic disease	1
Chronic lymphocytic leukemia	1	Proliferative diseases	1
Coronary heart disease	1	Prostate cancer (hormone refractory)	1
Dilated cardiomyopathy	1	Pseudohypoaldosteronism type I, autosomal dominant	1
Hyperkalemic distal renal tubular acidosis (RTA type 4)	1	Rheumatoid arthritis	1
Hyperlipidemia	1	Squamous cell carcinoma	1
Hypertension, early-onset, autosomal dominant, with exacerbation in pregnancy	1	Tremor, unspecified	1
Hypoglycemia	1	Waldenstrom's macroglobulinemia	1
Hypothermia	1		
